# Pattern of cytokines and chemokines in exhaled breath condensate is related to the characteristics of mechanical ventilation

**DOI:** 10.1186/cc9564

**Published:** 2011-03-11

**Authors:** I Van Walree, A Van Houte, G Rijkers, H Van Velzen-Blad, H Endeman

**Affiliations:** 1 Diaconessenhuis, Utrecht, the Netherlands; 2 St Antonius Hospital, Nieuwegein, the Netherlands

## Introduction

Ventilator-associated lung injury (VALI) is an inflammatory response of the lung caused by mechanical ventilation (MV) and is related to tidal volumes (TV), positive end-expiratory pressure (PEEP) and peak pressures (P_peak_) [1]. In experimental settings, VALI is characterized by a local inflammatory response measured in tissue or lavate. It is difficult to obtain this material in the critically ill [2]. Exhaled breath condensate (EBC) is obtained in patients on MV using an easy non-invasive technique. In this pilot study we examined the relation between levels of inflammatory proteins in EBC of patients on MV and characteristics of MV.

## Methods

A prospective study was performed in 13 patients on MV. EBC was obtained from the connection-swiffle between ventilator and tube. IL-1β, IL-4, IL-6, IL-8, IL-10, IL-12, IL-17, IFNγ, MCP-1 and MIP-1β were determined by multiplex immunoassay. Levels of inflammatory mediators were correlated with parameters of MV.

## Results

In 13 (seven males) patients, 29 samples were obtained. Median age of the patients was 69 years, median APACHE II score 25 points. Samples were taken during MV: seven during pressure control (PC) and 22 during pressure support (PS) mode. Median P_peak _was 18 cmH_2_O, median PEEP 8 cmH_2_O, median TV 7.22 ml/kg and median P/F ratio 33.62 kPa. Levels of all inflammatory proteins except for IL-12 were lower in patients on PC, reaching statistical significance for IL-17 (median PS 1.96 vs. PC 0.96, *P *= 0.002) and MCP-1 (median PS 0.72 vs. PC 0.38, *P *= 0.033). Significant lower levels were found in patients ventilated with TV ≤8 for MCP-1 (median TV ≤8 ml/kg 0.75 vs. TV >8 ml/kg 3.41, *P *= 0.032) and MIP-1β (median TV ≤8 ml/kg 0.00 vs. TV >8 ml/kg 1.30, *P *= 0.028). Levels of cytokines were lower in case of low P_peak _(≤20 cmH_2_O) reaching the level of statistical significance for IFNγ (median P_peak _≤20 cmH_2_O 0.00 vs. > 20 cmH_2_O 6.23, *P *= 0.025).

## Conclusions

In a small group of patients, cytokine and chemokine patterns in EBC were related with characteristics of MV. MV with a TV ≤8 may limit inflammatory response.
